# Exploration of the effects of the CYCLOPS gene *RBM17* in hepatocellular carcinoma

**DOI:** 10.1371/journal.pone.0234062

**Published:** 2020-06-04

**Authors:** Can Li, Shanghua Ge, Jialu Zhou, Jie Peng, Jiayu Chen, Shuhui Dong, Xiaofang Feng, Ning Su, Lunli Zhang, Yuanbin Zhong, Libin Deng, Xiaoli Tang

**Affiliations:** 1 Queen Mary School, Medical College of Nanchang University, Nanchang, China; 2 Jiangxi Provincial Key Laboratory of Preventive Medicine, School of Public Health, Nanchang University, Nanchang, China; 3 The Second Clinical College, Medical College of Nanchang University, Nanchang, China; 4 The Fourth Clinical College, Medical College of Nanchang University, Nanchang, China; 5 Department of Infectious Diseases & Key Laboratory of Liver Regenerative Medicine of Jiangxi Province, The First Affiliated Hospital of Nanchang University, Nanchang, China; 6 College of Basic Medical Science, Nanchang University, Nanchang, China; University of Alabama at Birmingham, UNITED STATES

## Abstract

**Background:**

Hepatocellular carcinoma (HCC) is one of the most lethal and malignant tumours worldwide. New therapeutic targets for HCC are urgently needed. CYCLOPS (copy number alterations yielding cancer liabilities owing to partial loss) genes have been noted to be associated with cancer-targeted therapies. Therefore, we intended to explore the effects of the CYCLOPS gene *RBM17* on HCC oncogenesis to determine if it could be further used for targeted therapy.

**Methods:**

We collected data on 12 types of cancer from the Cancer Genome Atlas (TCGA) and Gene Expression Omnibus (GEO) queries for comparison with adjacent non-tumour tissues. *RBM17* expression levels, clinicopathological factors and survival times were analysed. RNAseq data were downloaded from the Encyclopaedia of DNA Elements database for molecular mechanism exploration. Two representative HCC cell models were built to observe the proliferation capacity of HCC cells when *RBM17* expression was inhibited by sh*RBM17*. Cell cycle progression and apoptosis were also examined to investigate the pathogenesis of *RBM17*.

**Results:**

Based on 6,136 clinical samples, *RBM17* was markedly overexpressed in most cancers, especially HCC. Moreover, data from 442 patients revealed that high *RBM17* expression levels were related to a worse prognosis. Overexpression of *RBM17* was related to the iCluster1 molecular subgroup, TNM stage, and histologic grade. Pathway analysis of RNAseq data suggested that *RBM17* was involved in mitosis. Further investigation revealed that the proliferation rates of HepG2 (P = 0.003) and SMMC-7721 (P = 0.030) cells were significantly reduced when *RBM17* was knocked down. In addition, *RBM17* knockdown also arrested the progression of the cell cycle, causing cells to halt at the G2/M phase. Increased apoptosis rates were also found *in vitro*.

**Conclusion:**

These results suggest that *RBM17* is a potential therapeutic target for HCC treatment.

## Introduction

Hepatocellular carcinoma (HCC) is the sixth most frequent malignancy worldwide, with high mortality rates and poor prognosis [1]. Based on Cancer Today data (http://gco.iarc.fr/today/home), there were 841,080 new cases and 781,631 deaths related to HCC worldwide in 2018, accounting for 4.7% of the total cancer new cases. HCC incidence is very high in the Asia-Pacific region, remarkably so in China [2–4]. In the clinic, systemic therapies including chemotherapy, surgical excision, and liver transplantation are applied for most HCC patients [5]. Nevertheless, the five-year survival rate of surgical excision patients is 30%-50%, and the whole prognosis of HCC patients is poor [6–8]. Hence, further exploration of HCC treatment, especially targeted therapy, is urgently and crucially needed.

In contrast to normal cells, cancer cells are more dependent on pathways that eliminate cancer-related stressors that include DNA damage replication stress, proteotoxic stress, mitotic stress, metabolic stress, and oxidative stress [9]. Moreover, this state is created to promote cancer formation [10]. Genomic instability is a source of cancer-specific stress. This instability can lead to alteration of gene copy number. In previous research, copy number alteration was suggested to affect approximately 11% of the genome, including thousands of genes [11]. Part of the altered genes are driver genes, which directly activate oncogenes or inhibit suppressor genes. The remaining genes are not driver genes but are essential genes; they might lead to sensitivity to further gene suppression and then promote cancer cell stress [10].

Accordingly, these non-driver but essential genes could be targets for cancer therapy. William and his team utilized Affymetrix SNP 6.0 array data to rate the significance of the difference in copy-loss and copy-neutral classes of genes in a genome-wide study [11]. They identified 56 genes whose loss correlated with a high sensitivity to further gene suppression after normalizing and filtering from 3,131 tumours and 86 cell lines [12]. These genes were defined as CYCLOPS (copy number alterations yielding cancer liabilities owing to partial loss) genes. Functional analysis proposed 56 CYCLOPS genes with three classifications by assigning class labels, including spliceosome, ribosome, and proteasome KEGG pathway designations. However, only the biological function of *PSMC2* was explored, and whether the other genes are vital is not clear [13]. CYCLOPS genes tend to be a subset of essential genes with alteration caused by somatic copy number alterations. More CYCLOPS gene dependencies are associated with spliceosome components [14].

Comprehensively based on the previous research, *RBM17* is reported as a CYCLOPS gene that received the spliceosome KEGG pathway designation with great research value. *RBM17* (RNA binding motif protein 17, also known as *SPF45*) encodes an RNA binding protein, which is a component of the spliceosome complex involved in the second catalytic step of alternative splicing [15–17]. Alternative splicing is vital to regulate gene expression by generating more than one unique mRNA [18, 19]. Previous studies have shown that *RBM17 is* overexpressed in tumours, and its silencing can reduce hypopharyngeal carcinoma and glioma cell proliferation [20–22].

Here, we aspire to explore the influence of *RBM17* expression on cancer pathogenesis. We systematically collected expression data for *RBM17* in 12 types of cancer and 3 queries from the Cancer Genome Atlas (TCGA) and Gene Expression Omnibus (GEO) databases. After screening 12 types of cancer, *RBM17* was overexpressed most significantly in HCC. By means of integrated data analysis, we evaluated the effects of *RBM17* on prognosis and various clinical phenotypes and verified its influence on cell proliferation by constructing cytological models. Therefore, our purpose was to demonstrate that *RBM17* can be considered a potential therapeutic target for HCC, providing an innovative option for the treatment of HCC patients.

## Materials and methods

### Data extraction and analysis

To explore the *RBM17* expression level, we collected TCGA transcription data for 12 types of cancer, whose tumour and non-tumour sample sizes were both more than 20, including breast invasive carcinoma (BRCA), colon adenocarcinoma (COAD), head and neck squamous cell carcinoma (HNSC), kidney chromophobe carcinoma (KICH), kidney renal clear cell carcinoma (KIRC), kidney renal papillary cell carcinoma (KIRP), liver hepatocellular carcinoma (LIHC), lung adenocarcinoma (LUAD), lung squamous cell carcinoma (LUSC), prostate adenocarcinoma (PRAC), stomach adenocarcinoma (STAD), and thyroid carcinoma (THCA). Gene expression data and clinicopathological staging data of *RBM17* in 12 types of cancer were obtained from the TCGA database. We downloaded them from the cBioPortal for Cancer Genomics (www.cbioportal.org). For HCC, data for a total of 343 samples, including patient survival time, age, sex, histological staging, pathological staging, and iCluster, were downloaded and stored in the ".txt" format. The high expression group was defined as samples with an expression level of *RBM17* greater than the median, while the lower expression group was defined as samples with expression levels below the median. We classified, quantified and analysed the above TCGA data by using SPSS 22.0 software. χ^2^ analysis was applied for different clinical factors, and Kaplan-Meier survival curves were plotted. The expression levels of *RBM17* in different cancer types were also collected from TCGA. Meanwhile, we also performed a t-test to identify whether the difference in *RBM17* expression between these two groups was statistically significant.

In Gene Expression Omnibus (GEO), we searched the literature with three keywords: HCC, *Homo sapiens*, and expression profiling by an array. Three HCC-related GEO datasets were selected from 5 criteria: (1) samples ≥ 20; (2) sample source: tissue; (3) Affymetrix array platform; (4) file type:.CEL; (5) includes both tumour and non-tumour tissue. They were GSE6764, GSE45267, and GSE41804, including 184 samples with the specific probe 224781_s_at. GEO2R is an R package using both the GEOquery and limma packages to determine the difference in expression level between the cancerous tissue and noncancerous tissue sample groups.

We downloaded RNAseq data from six groups of HepG2 cells from the Encyclopaedia of DNA Elements (ENCODE) database to detect the effect of *RBM17* knockdown. Among these, four groups were normal control groups, and two groups were shRNA of *RBM17*. The original data were uploaded to Network Analyst (https://www.networkanalyst.ca). Matched genes between the control groups and shRNA groups were compared. After filtering and normalization, we obtained the differentially expressed genes (DEGs). To explore the specific molecular mechanism of DEGs in the occurrence and development of HCC, we conducted enrichment analyses of the most significant DEGs obtained. To identify the most significant DEGs, we used a web-based analysis tool (http://www.webgestalt.org) to acquire gene ontology (GO) annotations with a significance threshold of false discovery rate (FDR) less than 0.1.

### Cell culture

In the experimental investigation, eight cell lines were required: Hep3B, SKHEP-1, Huh7, HepG2, HCC-LM3, SMCC-7721, BEL-7402, and MHCC-97L. SKHEP-1 human cell lines were purchased from the American Type Culture Collection (Manassas, VA, USA). The HepG2, Hep3B, SMCC-7721, BEL-7402, Huh7, and HCC-LM3 cells were obtained from the Cell Bank of the Type Culture Collection of the Chinese Academy of Sciences (Shanghai, China). MHCC-97L was bought from Shanghai Biological Technology Co., Ltd. enzyme research (Shanghai, China). We cultured the eight strains of cells in DMEM containing 10% foetal bovine serum (Gibco) at 37°C with 5% CO2.

### RNA extraction and quantitative RT-PCR

Total RNA was extracted using TRIzol reagent (Invitrogen). For real-time PCR, we used 1 μg RNA for the reverse transcription reaction with the PrimeScript RT reagent kit with gDNA Eraser (Takara). Real-time fluorescence quantitative PCR was performed using a ViiA7 real-time PCR System. The primers used in the qRT-PCR were *RBM17* forward primer: 5'-AGAAGGCAGCTCTCACTCAG-3' and *RBM17* reverse primer: 5'-ATTTGCCGGTCATCTGAGGA-3'. We normalized the data to the housekeeping gene GAPDH. The primers for the housekeeping gene were as follows: GAPDH forward primer: 5'-TGACTTCAACAGCGACACCCA-3' and GAPDH reverse primer: 5'- CACCCTGTTGCTGTAGCCAAA-3'.

### *RBM17*-shRNA sequence construction and transfection

Cytological experiments analysed the role of *RBM17* in the development of HCC. Three sh*RBM17* RNA sequences were designed to construct plasmids. The shRNA sequences targeting *RBM17* were as follows: sh*RBM17*-1: caccGATGAAGCAGTACGGATATTTttcaagagaAAATATCCGTACTGCTTCATCttttttg; sh*RBM17*-2: caccGAGAAAGTAGTGAAGCGCCAAttcaagagaTTGGCGCTTCACTACTTTCTCttttttg; and sh*RBM17*-3: caccAGATGAAGATTATGAGCGAGAttcaagagaTCTCGCTCATAATCTTCATCTttttttg. We used *FoxM1* as positive control gene, and the positive control shRNA sequence was caccGCCAATCGTTCTCTGACAGAAttcaagagaTTCTGTCAGAGAACGATTGGCttttttg. To improve the efficacy of transient plasmid transfection of HCC cells, Lipofectamine 2000 (Invitrogen, Carlsbad, CA) was used. A scrambled shRNA was used as a negative control. We determined *RBM17* expression levels in cells after 48 h. Meanwhile, the oligonucleotides for shRNA expression were introduced into pGPU6 vector.

### Western blotting

Total protein was extracted using radioimmunoprecipitation lysis buffer Roche, Nutley, NJ, USA). Protein lysates were quantified with a BioRad kit (Biorad, Hercules, CA, USA). Microgram protein samples of SMMC-7721 cells were separated via 10% sodium dodecyl sulfate polyacrylamide gel electrophoresis (SDS-PAGE) and then electrophoretically transferred to a polyvinylidene difluoride membrane (Millipore, MA, USA). The membranes were immunoblotted with primary antibodies overnight at 4°C followed by respective secondary antibodies (horseradish peroxidase-conjugated goat anti-rabbit IgG antibody) incubating 4 hours. EasyBlot ECL kit is used to detect the band (Bio Basic Inc., Markham, ON, Canada). The antibodies used as follows: *RBM17* (1:500; cat. No. 101441; Abcam, Cambridge, UK); *GAPDH* (1:2,000, cat. No. sc‐32233; Santa Cruz Biotechnology, Inc., Dallas, TX, USA); horseradish peroxidase-conjugated goat anti-rabbit IgG antibody (1:10,000, cat. No. sc‐2005; Santa Cruz Biotechnology, Inc., Dallas, TX, USA).

### Cell proliferation assay

The constructed shRNA plasmid of *RBM17* was transfected into Huh7 and SMMC-7721 cells and set as a control group. Proliferation was measured according to the instructions of the BrdU colorimetric cell proliferation assay kit (cat# 1,647,229; Roche). In all experiments, HCC SMMC-7721 and Huh7 cells were first inoculated in 6-well plates at a density of 1×10^6^ cells per well. Next, the cells were serum-starved for 36 hours to induce the dormant phase. The cells were then re-stimulated with 10% (v/v) FBS for 18 hours before labelling the cells with BrdU for 2 hours. The number of BrdU-positive cells per 100 cells was expressed as the percentage of proliferating cells.

### Cell cycle and apoptosis detection

We collected HepG2 cells (2 × 10^6^) with or without sh*RBM17* treatment. Half of these cells were fixed with 500 μL 70% cold ethanol for 2 h overnight according to the manual for the cell cycle detection kit (Keygen Biotech Co., Ltd., Nanjing, Jiangsu, China) and stored at 4°C. After the removal of fixative with PBS, the cells were placed in a water bath, and 100 μL RNase A was added at 37°C and incubated for 30 min. Then, 400 μL propidium iodide (PI) was added to the cells and incubated for 30 min in the dark. The cell cycle was detected on a flow cytometer (BD Accuri C6, Ann Arbor, MI, USA) by red fluorescence set to a wavelength of 480 nm and analysed by FlowJo software.

The other half of the cells was reacted with 5 μL annexin V-fluorescein isothiocyanate (FITC) and 5 μL PI at room temperature in the dark for 5–15 min according to the instructions of a cell apoptosis detection kit (Keygen Biotech Co., Ltd., Nanjing, Jiangsu, China). A flow cytometer with FL1 and FL3 detectors was set to record at a wavelength of 480 nm.

### Statistical analyses

Data were expressed as mean ± standard deviation. Student's t‐test was performed to analyse differences between two groups using SPSS 22.0 software (SPSS Inc., Chicago, IL, USA). P<0.05 were considered statistically significant.

## Results

### *RBM17* is overexpressed in most cancers and significantly expressed in HCC

**Fig 1:**
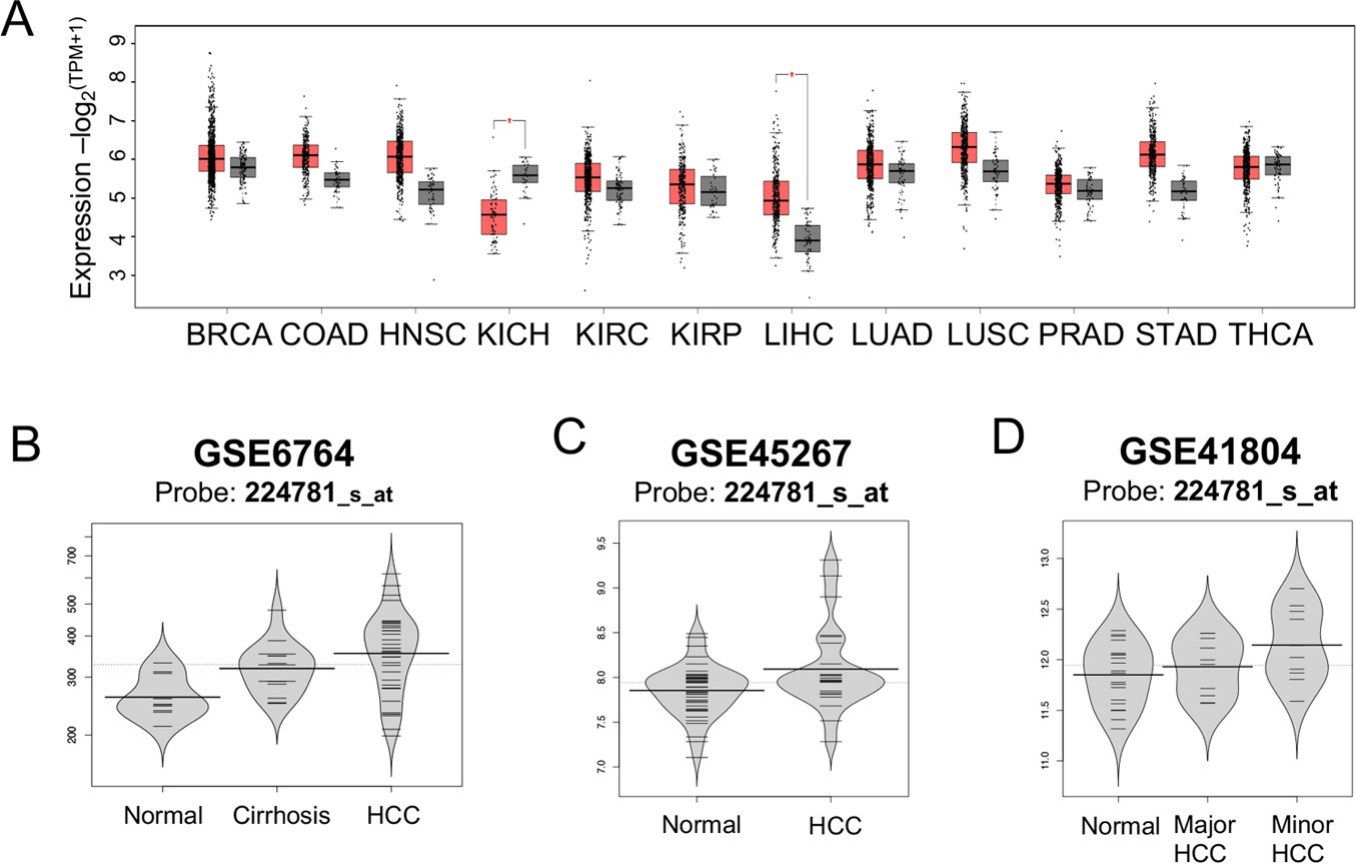
*RBM17* was highly expressed in tumour tissues, as verified by TCGA and GEO datasets. (A) A total of 6,136 samples were analysed in 12 cancers from TCGA, including 5,504 tumour tissue samples and 632 non-tumour tissue samples. *RBM17* was highly expressed in HCC and had low expression in kidney chromophobe tumours, with statistical significance (P < 0.05). (B) GSE6764 contained 23 normal tissue samples and 35 HCC cancer samples. *RBM17* was overexpressed in HCC samples (P = 0.005). Meanwhile, the expression of *RBM17* was higher in cirrhotic liver tissue than normal tissue. (C) For GSE45267, there were 41 normal tissues and 24 HCC tumour tissues. The violin plot revealed that the expression of *RBM17* was obviously high in HCC cancer tissue (P = 1.02x10^-9^). (D) In GSE41804, 40 samples consisted of 20 normal tissues, 10 major HCC tissues, and 10 minor HCC tissues. Thereinto, major HCC refers to a single tumor nodule with a diameter of more than 5 cm. In comparison, minor HCC refers to a single tumor nodules with a maximum diameter of no more than 3 cm or two tumor nodules with a total diameter of no more than 3 cm. The t-test results revealed that major HCC (P = 0.035) and minor HCC (P = 0.022) samples had significantly higher *RBM17* expression than normal tissue.

In total, the 12 types of cancer we assembled from TCGA transcription data contained 6,136 samples, including 5,504 tumour tissue samples and 632 non-tumour tissue samples. After comparing the expression level of the tumour and non-tumour tissues, the results indicated that *RBM17* was overexpressed in most cancers, and only HCC manifested significantly different expression (Fig 1A, P-value < 0.05). The expression values of 185 samples from three GEO queries also confirmed that *RBM17* expression in tumour tissues was higher than that in non-tumour tissues (Fig 1B, 1C and 1D).

### Overexpression of *RBM17* was associated with clinicopathological characteristics and poor prognosis

**Fig 2:**
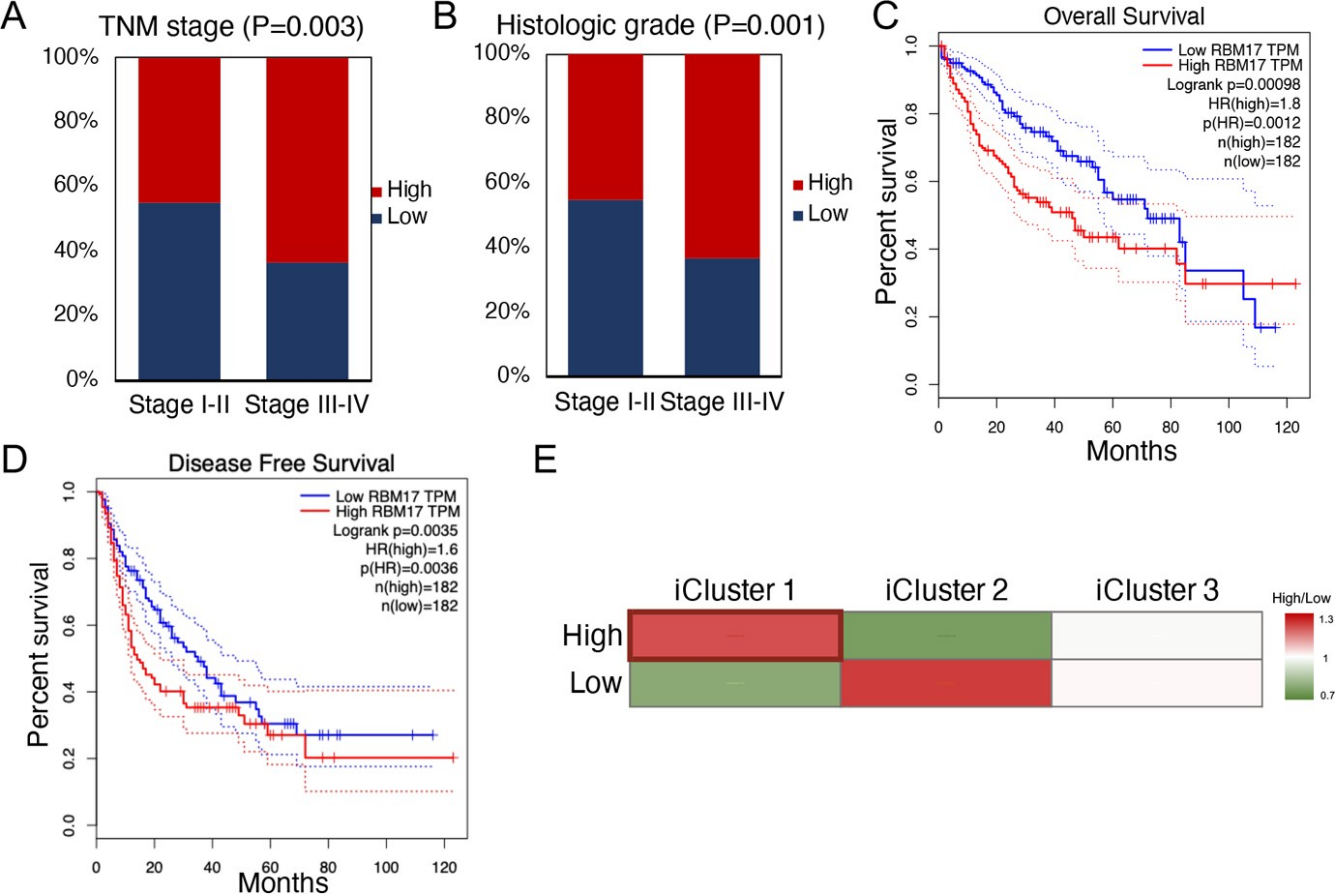
The association of *RBM17* expression and clinical characteristics. (A) The relationship between *RBM17* expression levels and HCC histologic grades was significantly correlated (P = 0.001). (B) *RBM17* overexpression was associated with the TNM stage (P = 0.003). (C) In the overall survival analysis, patients with high *RBM17* expression had shorter survival than patients with low *RBM17* expression (P = 0.001). (D) For disease-free patients, the ten-year survival rate of low *RBM17*-expressing patients was higher than that of the other patients (P = 0.004). (E) In iCluster1, most patients exhibited *RBM17* overexpression showing with border, while downregulation was more prominent in iCluster2. iCluster3 showed no difference. Based on the chi-square test, iCluster1 and the combination of the remaining two clusters were significantly correlated (P = 0.017).

According to the median expression level as the cut-off, we divided patients equally into two groups. Patients above the median were divided into “High” groups. Those below the median were divided into “low” groups. Primarily, we performed chi-square analysis of the two groups to explore the effect of *RBM17* overexpression on the clinicopathological characteristics of HCC (S1 Table). The results indicated that there was a tight correlation between the overexpression of *RBM17* and TNM grade and histologic stage (Fig 2A and 2B, P < 0.01). Furthermore, we analysed the expression level of *RBM17* in 364 patients with HCC to determine the connection between the expression level of *RBM17* and prognosis. The overall survival rates (P = 0.001) and disease-free survival rates (P = 0.004) revealed that high expression of *RBM17* was positively correlated with shorter survival time and worse prognosis in HCC (Fig 2C and 2D).

We additionally noticed that the expression of *RBM17* was related to the multiplatform integrative molecular typing of TCGA. We divided HCC patients with different phenotypes into three subgroups (iCluster1, iCluster2, and iCluster3), and subsequent analysis explored the relationship between the expression of *RBM17* and cell proliferation. After analysing the cluster data, we noticed that a large number of *RBM17*-overexpressing patients were gathered in iCluster1 (P = 0.017, Fig 2E). The results revealed that high expression of *RBM17* was positively correlated with the increment. Moreover, the aggregation of patients with high expression in iCluster1 suggested that *RBM17* expression is related to cell proliferation.

### Effect of *RBM17* knockdown on the proliferation of HCC cell lines

**Fig 3:**
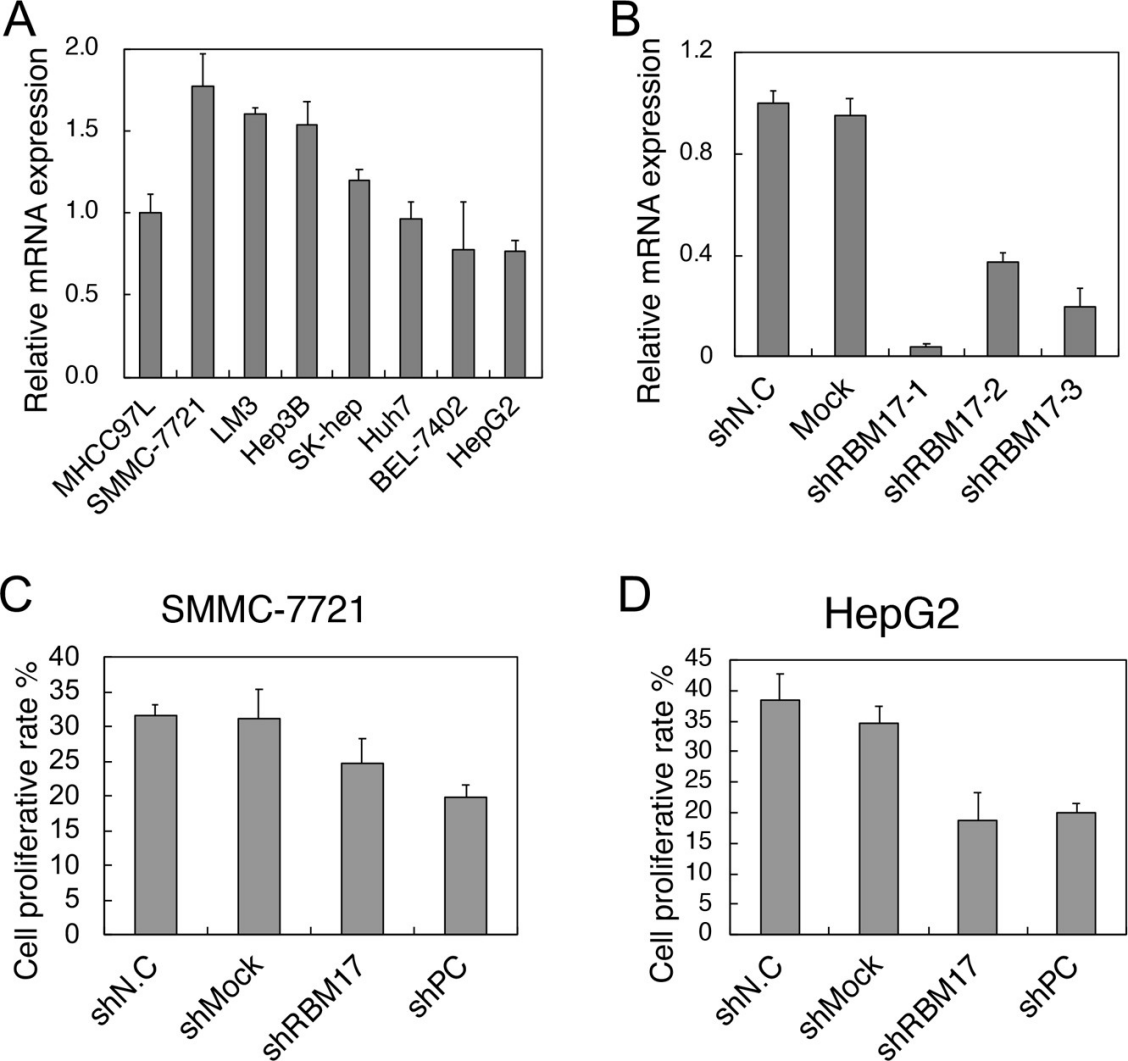
Cell proliferation rates of different cell lines with control and sh*RBM17* plasmids. (A) Among eight cell lines, SMMC-7721 had the highest expression level, while HepG2 had the lowest. (B) We detected expression levels of three constructed plasmids in SMMC-7721 cell line. sh*RBM17*-1 had the most significant effect with the lowest expression level among three sh*RBM17* plasmids, a mock plasmid, and a negative control plasmid. (C and D) sh*RBM17*-1, shPC (*FoxM1*), shNC, and shMock were transfected into SMMC-7721 and HepG2 cell lines. After knockdown, HepG2 cell proliferation was reduced in both the SMMC-7721 cell line (P = 0.030) and the HepG2 cell line (P = 0.003).

We conducted a series of experiments in 8 HCC cell lines (MHCC-97L, SMMC-7721, LM3, Hep3B, SK-HEP, Huh7, BEL-7402, and HepG2) to investigate the cellular role of *RBM17* in HCC oncogenesis. Two representative cell lines (SMMC-7721 and HepG2) were selected for subsequent experiments. The SMMC-7721 cell line showed the highest expression, while low expression was found in the HepG2 cell line (Fig 3A). Three sh*RBM17* RNA plasmids were constructed. We evaluated their gene silencing capabilities by qRT-PCR in SMMC-7721 cell line, which expressed the highest. sh*RBM17*-1 most potently decreased *RBM17* expression (Fig 3B) and was successfully constructed and transfected instantaneously. The protein levels of these three shRNAs detecting by WB also confirmed that sh*RBM17*-1 had the most significant effect on *RBM17* knocking down (S1 Fig). Meanwhile, control plasmids were also transfected into these two cell lines. After transfection for 48 h, cells transfected with sh*RBM17*-1 manifested a lower cell proliferation rate in both the SMMC-7721 cell line (P = 0.030, Fig 3C) and the HepG2 cell line (P = 0.003, Fig 3D). In summary, *RBM17* silencing can inhibit cell proliferation.

### *RBM17* knockdown disturbed the expression of cell cycle-related genes

**Fig 4:**
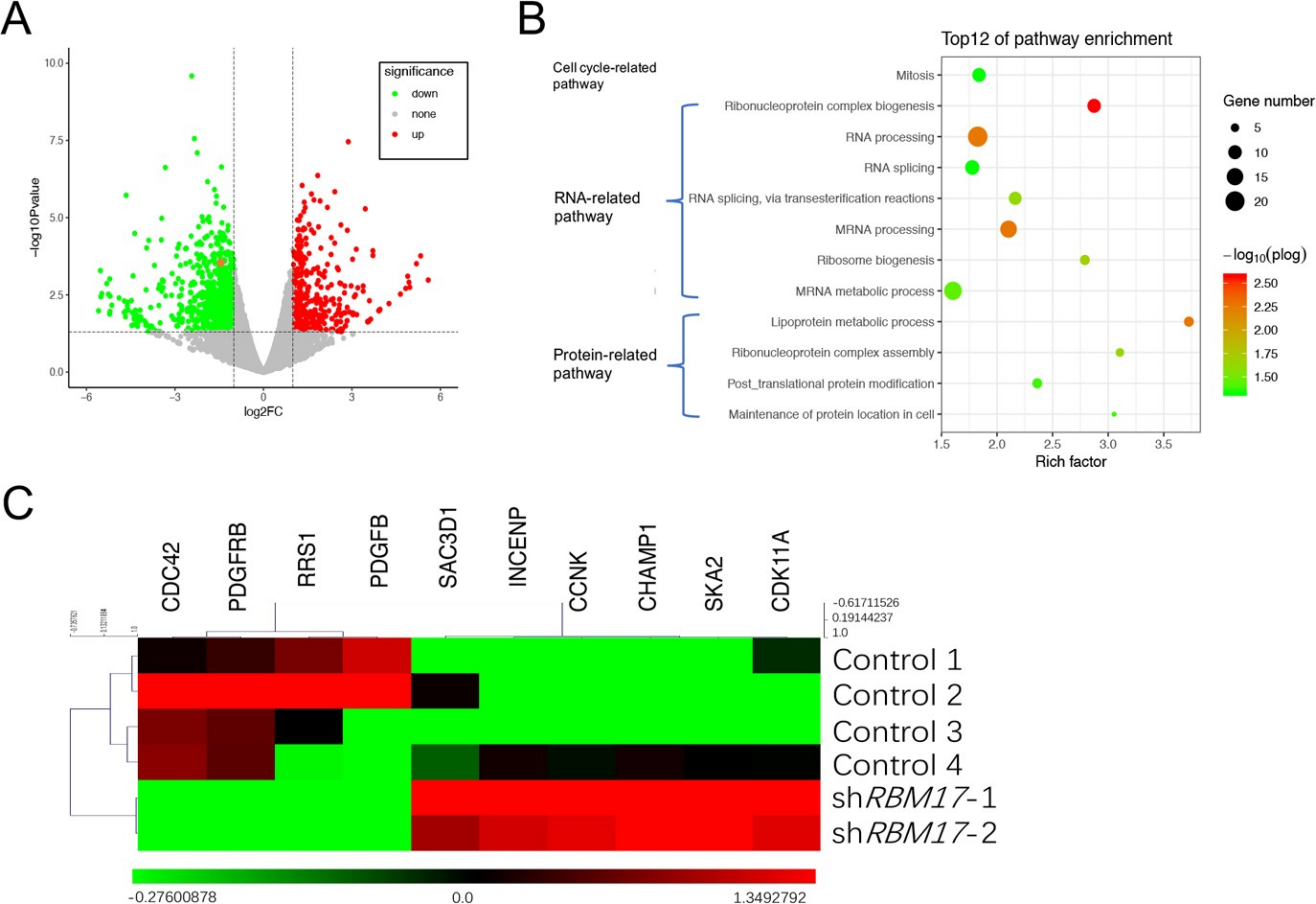
DEGs identification and pathway analysis. (A) The volcano plot for 1,106 significantly DEGs. There were 381 genes expressed at higher levels after *RBM17* knockdown. In contrast, 725 genes showed lower expression. We verified the accuracy by *RBM17* (orange spot). (B) Negative log base 10 Q-values from the pathway enrichment analysis are plotted, and P values of DEG-enriched pathways are sorted. Seven pathways were RNA-related, four pathways were protein-related, and one pathway was related to the cell cycle. (C) Ten core DEGs were involved in the mitosis pathway. A heat map of 10 core DEGs related to mitosis is plotted.

To further investigate the impact of *RBM17* on HCC development, RNAseq data was downloaded from ENCODE for analysis, including the two groups of sh*RBM17* cells and the four groups of the normal control cells in the HepG2 cell line. In line with the original data uploaded to Network Analyst network for differential expression analysis, there were 15,690 genes paired between the control groups and sh*RBM17* groups (Fig 4A). Genes with an FDR < 0.05 were considered DEGs induced by sh*RBM17*. Of the 1,106 DEGs, 381 genes (34.4%) were up-regulated and 725 gene (65.6%) were down-regulated.

Among the 1,106 DEGs, we selected the 266 most significant DEGs (|Log_2_FC| > 1). GO enrichment pathway analysis was applied to detect their potential role and provided directions for future research. We obtained twelve pathways according to the p-value (Fig 4B). Based on the names of the pathways, the 12 pathways prominently clustered in three groups: seven were RNA-related, four were protein-related, and one was mitosis-related. The mitosis pathway provided a good clue. We identified 10 DEGs (7 up-regulated DEGs: *INCENP*, *CCNK*, *RRS1*, *SAC3D1*, *CHAMP1*, *SKA2*, *CDK11A*; 3 down-regulated DEGs: *CDC42*, *PDGFB*, *PDGFRB*) in this pathway. The results of the pathway analysis included the cell cycle pathway, which showed that the ten DEGs were in this pathway. We speculated that these ten DEGs might be the potential downstream genes of *RBM17* affecting the cell cycle (Fig 4C). Therefore, the results of the pathway analysis indicated that *RBM17* expression might be related to the cell cycle process.

### *RBM17* silencing arrested the cell cycle

**Fig 5:**
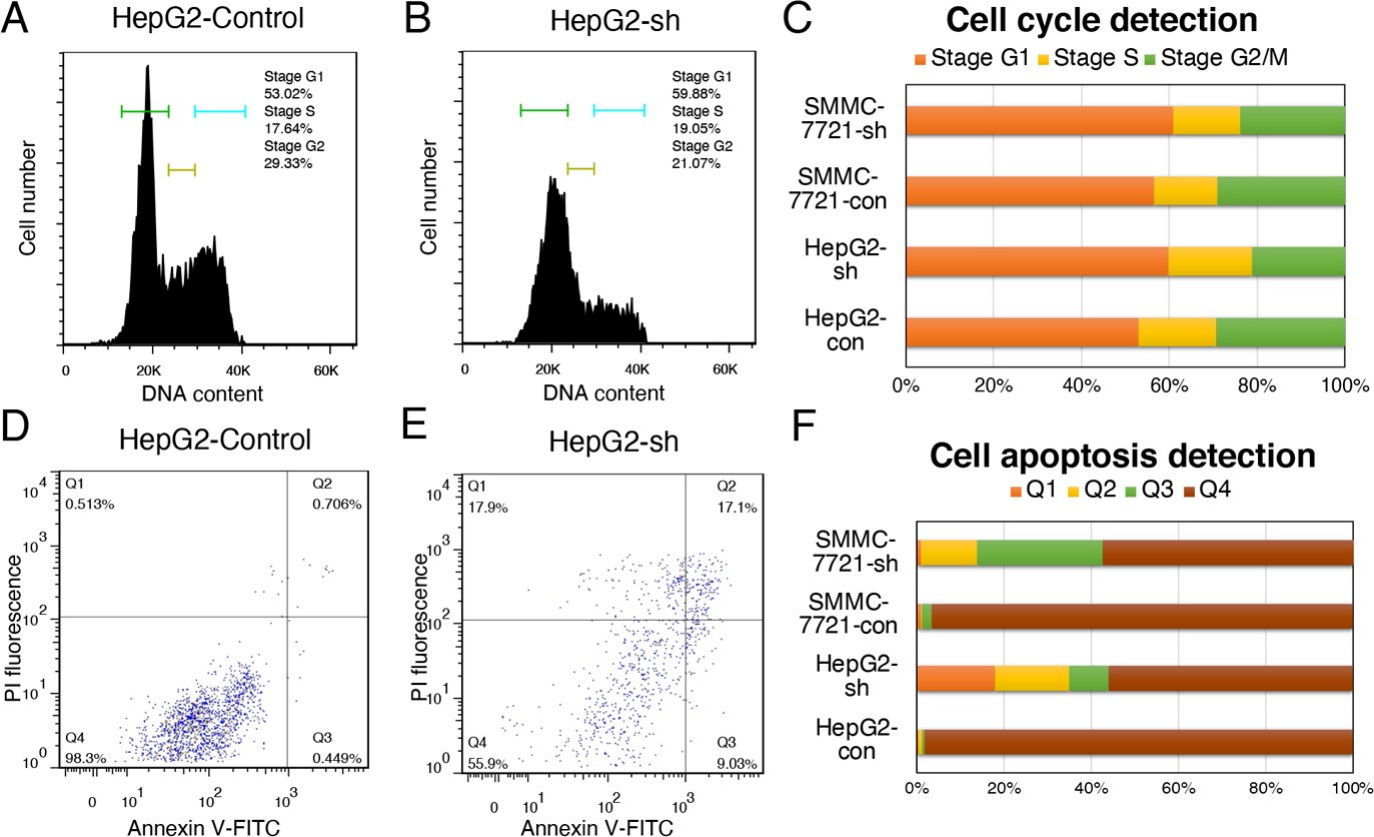
The effect of *RBM17* silencing on cell cycle progression and apoptosis. (A and B) We detected the cell cycle alteration after *RBM17* knocking down by flow cytometry. A showed the control group of cell cycle condition. Stage G1 occupied 53.020%, stage S occupying 17.640%, and stage G2/M was 29.330%. B showed the results of sh*RBM17* group. Stage G1 occupied 59.880%, stage S occupying 19.050%, and stage G2/M occupied 21.070%. Comparing to the control group, the cells of sh*RBM17* group arrested in G1 phase and shrunk in the G2/M phase. (C) The combination of the cell cycle detection results of HepG2 and SMMC-7721. In SMMC-7721 control group, stage G1 occupied 56.530%, stage S occupying 14.460%, and stage G2/M was 29.020%. Meanwhile, stage G1 occupied 60.960%, stage S occupying 15.260%, and stage G2/M was 23.780% in SMMC-7721 sh*RBM17* group. (D) We detected the apoptosis experiment on HepG2 cell line by flow cytometry. The abscissa and ordinate of the figures are the amounts of the Annexin V-FITC and PI, respectively. The sum of the fluorescence amount ratios occupied by Q2 and Q3 to infer the relative cell numbers of early and late apoptosis. In the control group, the percentages of cells in Q2 and Q3 were 0.706% and 0.449%, respectively. The sum of Q2 and Q3 was 1.155%. (E) In sh*RBM17*-1-transfected HepG2 cells, Q2 occupied 17.100%, while Q3 occupied 9.030% of the total cells. Moreover, the sum of the two was 26.130%. (F) The combination of the apoptosis detection results of HepG2 and SMMC-7721. In the control group of SMMC-7721, the percentages of cells in Q2 and Q3 were 0.256% and 2.200%, respectively. The sum of Q2 and Q3 was 2.456%. In sh*RBM17*-1 transfected SMMC-7721 cells, Q2 occupied 12.700%, while Q3 occupied 57.400% of the total cells. The sum of the two was 70.100%.

To explore the effect of *RBM17* on the cell cycle of HCC, we performed cellular experiments on both HepG2 and SMMC-7721 cell lines. HepG2 cells presented the most obvious inhibition of cell proliferation after *RBM17* knockdown. Therefore, HepG2 cells were transfected with sh*RBM17*-1 for 48 h. Meanwhile, empty plasmids were also transfected as a control. The flow cytometry results revealed that most cells arrested after *RBM17* knockdown, and more cells arrested in the G1 phase and shrunk in the G2/M phase after *RBM17* knockdown (Fig 5A and 5B). We also performed an apoptosis test. A strong inhibitory effect was observed in sh*RBM17*-1-transfected HepG2 cells with a distinct increase in the rates of early and late apoptosis Because the sum of relative death cells in Q2 and Q3 was obviously larger after *RBM17* knocking down, which reflected sh*RBM17* accelerating both early and late apoptosis (Fig 5D and 5E). The results of SMMC-7721 cell line were consistent with the results obtained by HepG2 before (Fig 5C and 5F). In general, high *RBM17* expression in HCC cells might be crucial in promoting cancer cell proliferation and preventing apoptosis.

## Discussion

There has been no research on the relationship between *RBM17* and HCC. We innovatively explore the clinical and molecular effects of *RBM17* in HCC to provide some clues for the future HCC treatment. The CYCLOPS genes contain several characteristics, which *RBM17* matches. CYCLOPS genes tend to be cell essential genes, and each CYCLOPS candidate exhibited copy number variation in 8% -34% of samples. The copy number variation exists in the tumor cells, which are bidirectional with both increasing and decreasing [14]. According to previous studies, *RBM17* is an essential gene that encodes the spliceosome complex component and participates in the alternative splicing process [15–17]. TCGA data shows that the expression level of *RBM17* in HCC patients is proportional to the copy number. The copy number variation of *RBM17* accounted for 28% of the sample, including both amplification and loss (S2A Fig). We acquired the cancer cell lines data from Cancer Cell Line Encyclopedia (CCLE). Many cell lines had copy number variations. Meanwhile, an increase in the copy number of *RBM17* leaded to an increase in expression, which is beneficial for oncogenesis (S2B Fig). However, it was found in our cytological experiments that HepG2 cell line expression was the lowest and SMMC-7721 the highest. But the subsequent proliferation experiments revealed that the lowest-expressing HepG2 cell line was the deadest.

Prior to the proposition of CYCLOPS, the conventional concept was to find a gene that was highly expressed in tumor cells, and that was associated with worsen effect on tumor progression. This gene can then be used as a target to treat tumors with drugs that knock it down. But the CYCLOPS gene proves that after knocking down the gene, cells with lower expression level are more likely to die. Therefore, this leads to a new way of treating cancer, which is to find low-expression cell lines to knock down. HepG2 and SMMC-7721 proliferation experiments proved this new way. And *RBM17* is also proved as a CYCLOPS gene. From a clinical perspective, if there is a drug that knocks down this gene, the marker for choosing suitable patients is the lower expression.

Although we detected that *RBM17* had a notable effect on the cell cycle and apoptosis in HCC, we did not carry out experimental verification on the specific influence of *RBM17* on HCC development [20]. The pathway enrichment analysis revealed that ten genes were associated with the cell cycle pathway. After matching with known information, we determined that *CDC42* [23, 24], *CDK11A* [25–27], *SAC3D1* [28–30], *CCNK* [31], and *INCENP* [32–34] were involved in mitosis and apoptosis progression and might be potential downstream targets by which *RBM17* can regulate cell proliferation in the development and progression of HCC.

*CDC42* has been demonstrated that its deregulation can change normal cell function like cell division; it can cause a tumor. In a typical case, maintain the expression level of *CDC42* can restrain oncogenesis [24]. *CDK11A* is positioned in the cytoplasm and nucleus and is expressed explicitly in the G2/M phase [25]. Abnormal expression of *CDK11A* can halt the cell cycle [27]. *SAC3D1* is acknowledged as a novel prognostic marker of HCC [28]. Centrosomes are vital structures that promote cell cycle progression and stabilize genomes [29]. *SAC3D1* may affect the progression of the cell cycle and promote the oncogenesis of HCC. *INCENP* (inner centromere protein) encodes a protein which contributes to activation of the mitotic checkpoint [33]. *INCENP* SAH domain's capacity of binding microtubules is crucial to the stagnation of mitosis, and further determines cell death rates [34].

Gitte and his team used the expressed sequence tag (EST) database and nanoelectrospray mass spectrometry to characterize *RBM17* as a DNA damage repair protein [35]. Therefore, we highly suspect that the splicing function of *RBM17* actually affects DNA repair, which in turn promotes cell cycle arrest. In summary, the exact molecular mechanism of *RBM17* in oncogenesis is still unclear and needs to be further explored.

## Supporting information

S1 Fig (TIF)

S2 Fig (TIF)

S1 Table (PDF)

S1 File (DOCX)

S1 Raw Images (PDF)
