# Supplementary figures and images for: Exploration of the effects of the CYCLOPS gene RBM17 in hepatocellular carcinoma
Source: PLoS One. 2020 Jun 4;15(6):e0234062. doi: 10.1371/journal.pone.0234062 (PMC7272028; doi:10.1371/journal.pone.0234062)

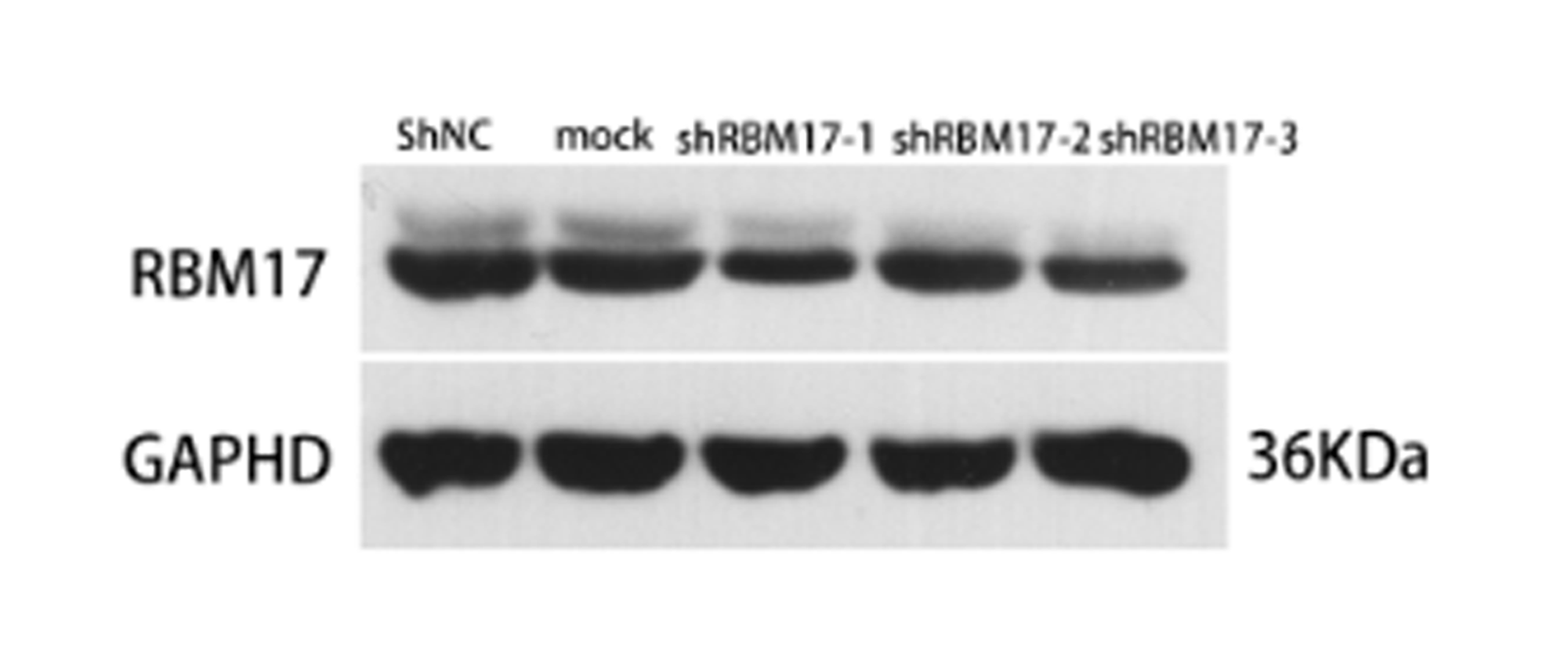

Supplement: S1 Fig — (TIF) [file pone.0234062.s001.tif]

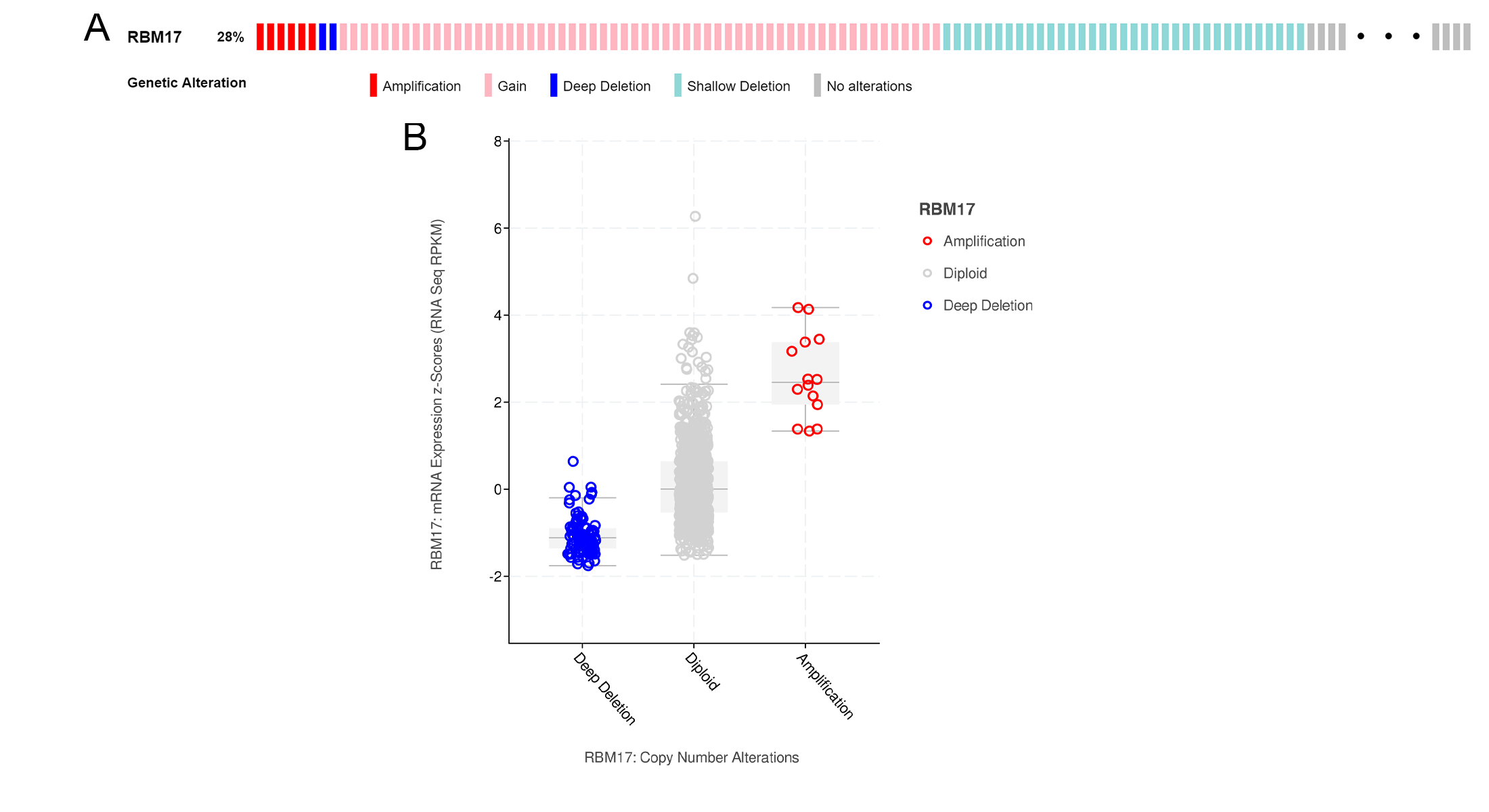

Supplement: S2 Fig — (TIF) [file pone.0234062.s002.tif]

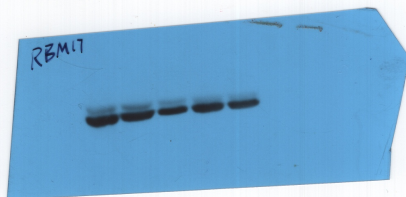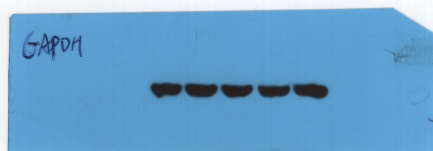

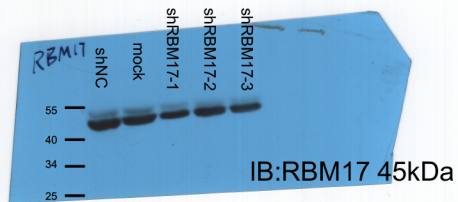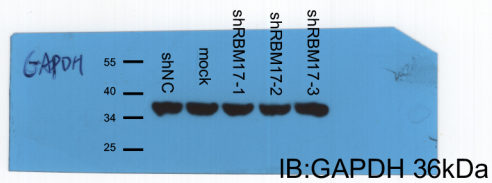

Supplement: S1 Raw Images — (PDF) [file pone.0234062.s005.pdf]
