# Supplementary material for: Exploration of the effects of the CYCLOPS gene RBM17 in hepatocellular carcinoma
Source: PLoS One. 2020 Jun 4;15(6):e0234062. doi: 10.1371/journal.pone.0234062 (PMC7272028; doi:10.1371/journal.pone.0234062)
Supplement: S1 Table — (PDF) [file pone.0234062.s003.pdf]

Supporting information

S1 Table. Association of *RBM17* expression levels with clinicopathologic characteristics in HCC (TCGA cohort)

Clinicopathologic Measurement data	*RBM17* expression (n=343)				X^2	P value
	High (n=171)		Low (n=172)			
	Count	%	Count	%		
Age						
≤50	46	13.45	30	8.77	4.448	0.038
>50	125	36.55	142	41.52		
Sex						
Male	108	31.58	125	36.55	3.565	0.065
Female	63	18.42	47	13.74		
TNM Stage						
Stage I-II	114	33.33	139	40.64	8.868	0.003
Stage III-IV	57	16.67	33	9.65		
Histologic Grade						
G1-G2	89	26.02	125	36.55	15.549	0.001
G3-G4	82	23.98	47	13.74		
