# Supplementary material for: Exploration of the effects of the CYCLOPS gene RBM17 in hepatocellular carcinoma
Source: PLoS One. 2020 Jun 4;15(6):e0234062. doi: 10.1371/journal.pone.0234062 (PMC7272028; doi:10.1371/journal.pone.0234062)
Supplement: S1 File — (DOCX) [file pone.0234062.s004.docx]

**Information about cell lines**

**Cell line sources**

1. SMMC-7721, HepG2, BEL-7402, and Hep3B, Huh7, HCC-LM3: were bought from the Cell Bank of the Type Culture Collection of the Chinese Academy of Sciences (Shanghai, China). http://www.cellbank.org.cn/index.asp
2. MHCC97L was bought from Shanghai Biological Technology Co., Ltd. enzyme research (Shanghai, China) in May 2018. http://www.elisakits.cn/Index/index.html
3. SKHEP-1 were brought from the American Type Culture Collection (Manassas, VA, USA). https://www.atcc.org

**Authentication:**

All the cell lines were not re-authenticated.

**Mycoplasma contamination:**

All the cell lines were tested with DNA stain for mycoplasma contamination and free of contamination.
